# Symmetry-protected ideal Weyl semimetal in HgTe-class materials

**DOI:** 10.1038/ncomms11136

**Published:** 2016-04-01

**Authors:** Jiawei Ruan, Shao-Kai Jian, Hong Yao, Haijun Zhang, Shou-Cheng Zhang, Dingyu Xing

**Affiliations:** 1 National Laboratory of Solid State Microstructures, School of Physics and Collaborative Innovation Center of Advanced Microstructures, Nanjing University, Nanjing 210093, China; 2 Institute for Advanced Study, Tsinghua University, Beijing 100084, China; 3 Collaborative Innovation Center of Quantum Matter, Beijing 100084, China; 4 Department of Physics, Stanford University, Stanford, California 94305, USA

## Abstract

Ideal Weyl semimetals with all Weyl nodes exactly at the Fermi level and no coexisting trivial Fermi surfaces in the bulk, similar to graphene, could feature deep physics such as exotic transport phenomena induced by the chiral anomaly. Here, we show that HgTe and half-Heusler compounds, under a broad range of in-plane compressive strain, could be materials in nature realizing ideal Weyl semimetals with four pairs of Weyl nodes and topological surface Fermi arcs. Generically, we find that the HgTe-class materials with nontrivial band inversion and noncentrosymmetry provide a promising arena to realize ideal Weyl semimetals. Such ideal Weyl semimetals could further provide a unique platform to study emergent phenomena such as the interplay between ideal Weyl fermions and superconductivity in the half-Heusler compound LaPtBi.

## Abstract (web summary)

Ideal Weyl semimetals, similar to graphene, show peculiar features such as exotic electronic transport. Here, Ruan *et al*. predict that strain-tuned HgTe-class materials can become ideal Weyl semimetals, which could provide a promising platform to study emergent topological phenomena.

## Graphical abstract

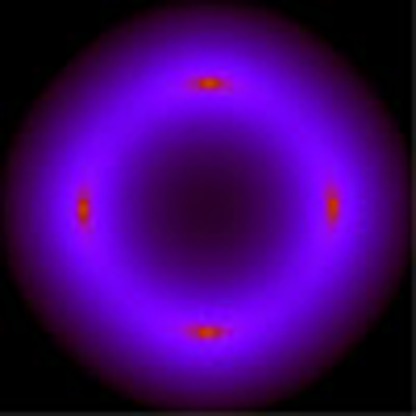

Weyl fermions were originally introduced as elementary particles in high energy physics more than 80 years ago^1^. While evidences of Weyl fermions as elementary particles remain elusive, realizing them as low-energy quasiparticles in solids has recently attracted increasing interest^2^,^3^,^4^,^5^,^6^,^7^,^8^,^9^,^10^,^11^,^12^,^13^,^14^,^15^,^16^,^17^,^18^, especially after the discovery of topological insulators^19^,^20^. In solids, Weyl nodes arise as discrete band-crossing points in crystal momentum space near the Fermi level, and Weyl fermions, as quasiparticles with linear dispersions across the Weyl nodes, can be described by the massless Dirac equation^21^. Weyl nodes carry a left- or right-handed chirality and they always appear in pairs of opposite chiralities according to the no-go theorem^21^. Intriguingly, it has been shown that the chiral anomaly in Weyl semimetals induces many manifestations in transport properties^21^,^22^,^23^,^24^,^25^,^26^,^27^,^28^,^29^,^30^ such as negative magnetoresistance, anomalous Hall effect and chiral magnetic effects. Another important hallmark of Weyl semimetals is its topologically protected unusual surface states—surface Fermi arcs^2^.

To realize Weyl semimetals in solids, either the time-reversal or lattice inversion symmetry has to be broken^2^,^3^,^4^,^5^,^6^,^7^,^8^,^9^,^10^,^11^,^12^,^13^. Recently, Weyl nodes were experimentally observed in noncentrosymmetric TaAs-class materials^14^,^15^,^16^,^17^,^18^. It was reported^14^,^15^,^16^ that these compounds host 24 Weyl nodes which are close to but not exactly at the Fermi level due to the fact that not all Weyl nodes are symmetry related. Moreover, there are also trivial Fermi pockets at the Fermi level in addition to the Weyl nodes. It is thus desirable to search for ideal Weyl semimetals with all Weyl nodes exactly residing at the Fermi level at stoichiometry composition (as shown in Fig. 1a) considering that ideal Weyl semimetals could maximize the potential for transport phenomena induced by the chiral anomaly and that supersymmetry may emerge at the pair-density-wave quantum criticality in an ideal Weyl semimetal^31^.

In this work, we show that the HgTe-class materials with both band inversion and lattice noncentrosymmetry, including mercury chalcogenide HgX (X=Te, Se)^32^ and the multifunctional half-Heusler compounds^33^,^34^,^35^, can realize symmetry-protected ideal Weyl semimetals with four pairs of Weyl nodes under a broad range of in-plane biaxial compressive strain. Note that strain engineering has been successfully employed in condensed matter systems to realize many physics^36^,^37^, which makes the experimental realization of strained HgX and half-Heusler compounds highly feasible. For simplicity, we focus on HgTe to discuss concrete results and to illustrate the general guiding principle to realize ideal Weyl semimetals in such class of materials.

## Results

### The effective model of HgTe

**Figure 1:**
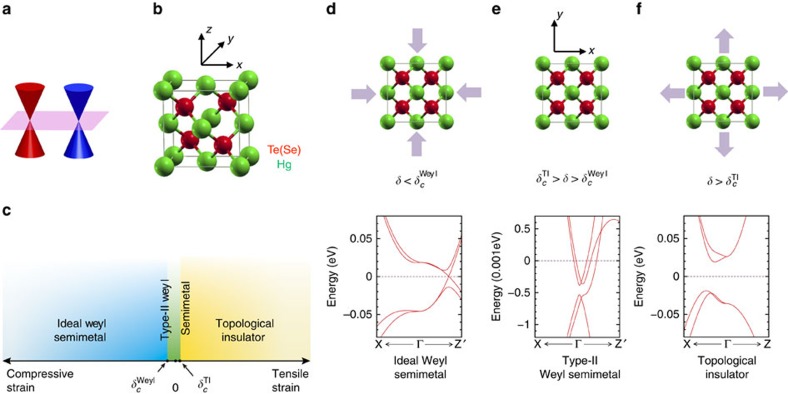
Phase diagram of strained HgTe. (**a**) The Weyl nodes in ideal Weyl semimetals are exactly at the Fermi level without coexisting trivial Fermi pockets. (**b**) The schematic representation of HgTe lattice with a typical zinc-blende structure. (**c**) For a certain range of strain in the *xy* plane, HgTe has three phases: ideal Weyl semimetals, type-II Weyl semimetals and topological insulators. (**d**) When the compressive strain is large enough 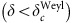, HgTe turns into the ideal Weyl semimetal phase. At stoichiometry, all Weyl nodes are exactly at the Fermi level. (**e**) When the strain is small enough 
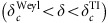
, HgTe is in the type-II Weyl semimetal phase. (**f**) When the tensile strain is large enough 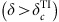, HgTe turns into the topological insulator phase.

HgTe has a typical zinc-blende structure with the space group 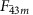 (Fig. 1b) and it is known to have an inverted band structure^32^ such that it is a semimetal and its Γ_8_ bands are half-filled. The Γ_8_ bands at the Γ point are fourfold degenerate as *J*=3/2 multiplet. Another important feature of HgTe is its bulk inversion asymmetry (BIA), which plays an essential role in realizing ideal Weyl fermions in strained HgTe as we shall show below. The Γ_8_ bands around the Γ point can be effectively described by the following Luttinger Hamiltonian^38^ plus perturbations due to the BIA:


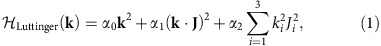


where *J*_*i*_ are spin-3/2 matrices and *α*_*i*_ are constants characterizing the band structure. The main perturbations induced by BIA are given by 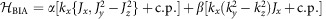, where {} represents anti-commutator, c.p. means cyclic permutations, and *α*,*β* are constants characterizing the strength of BIA^39^,^40^. By fitting the first-principle band structures around the Γ point, parameters above can be determined: for HgTe, *α*_0_≈109.8 Å^2^ eV, *α*_1_≈−45.87 Å^2^ eV, *α*_2_≈−19.73 Å^2^ eV and *α*≈0.208 Å^2^ eV.

Because of the BIA, dispersions around the Γ point are not purely quadratic and the Fermi level at stoichiometry is slightly above the fourfold degeneracy energy of the Γ_8_ bands^41^. Consequently, there exist tiny electron and hole pockets at the Fermi level. The existence of small Fermi pockets is further indicated by the line crossings between two intermediate bands protected by the crystalline symmetries of the cubic HgTe (Supplementary Fig. 1; Supplementary Note 1). Breaking the crystalline symmetry from T_*d*_ to D_2*d*_ by an in-plane strain can remove the line crossings and render the realization of ideal Weyl semimetals generically inevitable as we show below.

Strain in the *xy* plane generates a perturbation 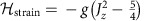, where *g* depends on the strength of strain and the lattice constant in the *xy* plane changes to *a*=(1+*δ*)*a*_0_. As explained in Supplementary Note 1, *g*>0 (*g*<0) for tensile strain *δ*>0 (compressive strain *δ*<0). The in-plane strain reduces the original cubic symmetry T_*d*_ to the tetragonal symmetry D_2*d*_ in which only two mirror planes (the *k*_*x*_=±*k*_*y*_ planes) survive and the other four are broken. As a result, line crossings originally protected by the broken mirror symmetries split except at discrete points in the *k*_*x*_=0 or *k*_*y*_=0 plane. The crossing points in the *k*_*x*_=0 or *k*_*y*_=0 plane are robust because these planes respect a special symmetry C_2*T*_=C_2_·*T*, where C_2_ is the two-fold rotation along the *x* or *y* axis and *T* is the time-reversal transformation (see Supplementary Note 1 for details). In total, there are eight Weyl nodes with four in the *k*_*x*_=0 or *k*_*y*_=0 plane, respectively.

### The phase diagram of strained HgTe

**Figure 2:**
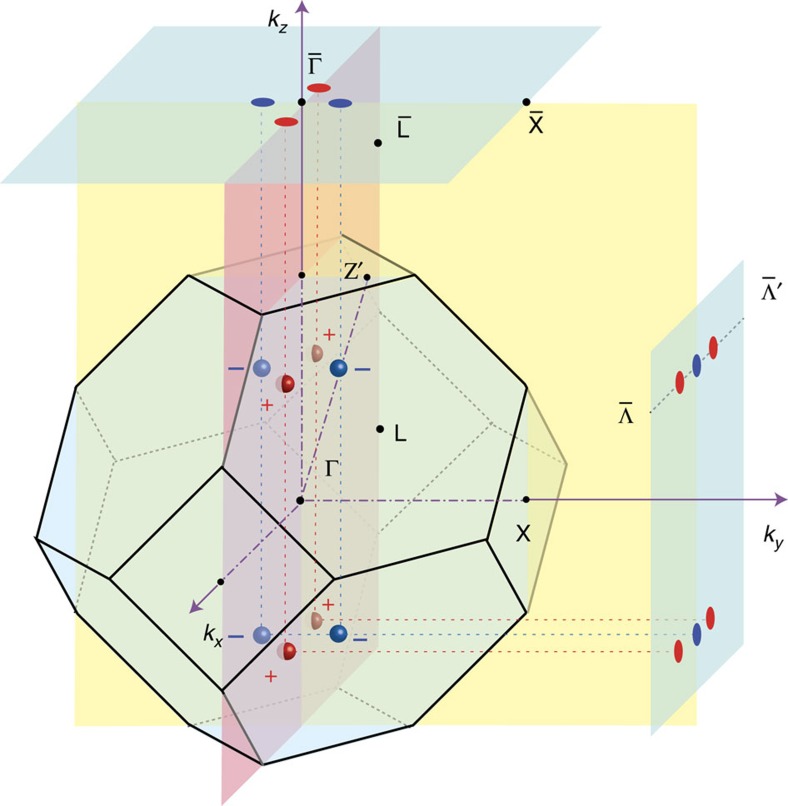
Schematic of Weyl points in Brillouin zone. The bulk Brillouin zone (BZ), and (001) and (010) surface BZs of HgTe. In the ideal Weyl semimetal phase of the strained HgTe, there are four pairs of ideal Weyl nodes in the bulk BZ, schematically shown as red (chirality +1) and blue (chirality −1) circles. The pink (yellow) plane is for the *k*_*y*_=0 (*k*_*x*_=0) plane. The projections of bulk Weyl nodes onto the (001) and (010) surface BZs are shown; there are four gapless points in the (001) surface BZ but six gapless points in the (010) surface BZ.

Under a sufficiently small strain, these Weyl nodes are type-II (ref. 13; see also Supplementary Fig. 2); they are close to but not exactly at the Fermi level and there are also other coexisting trivial Fermi pockets. Note that tensile and compressive strains have qualitatively different effect in moving these type-II Weyl nodes. Under a large enough tensile strain, the eight Weyl points annihilate with each other in the *k*_*x*_=±*k*_*y*_ planes, leading to a strong topological insulator, as shown in Fig. 1f, which agrees with previous theoretical calculations^40^ and experimental observations^42^. Intriguingly, under an increasing compressive strain, these type-II Weyl nodes quickly evolve to type-I Weyl nodes, shown in Fig. 1d, all of which lie exactly at the Fermi level leading to an ideal Weyl semimetal at stoichiometry, similar to graphene! Based on the **k·p** Hamiltonian 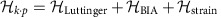, we obtain the whole phase diagram, schematically shown in Fig. 1c, where the strained HgTe is a strong topological insulator when 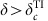, and is an ideal Weyl semimetal when 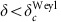. In the ideal Weyl semimetal phase, HgTe has eight Weyl nodes at 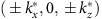 and 
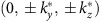
, as schematically shown in Fig. 2.

### The analysis of topological properties

Now, we use the **k·p** theory to analyse topological properties of ideal Weyl nodes. We first consider the effect of the strain and treat the BIA as a perturbation. It is straightforward to show that 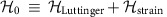 hosts two Dirac points in the *k*_z_ axis. The BIA perturbation splits each Dirac point into four Weyl nodes. The effective Hamiltonian around one of the Weyl points 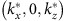 is described by the Weyl equation:


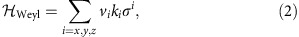


where *v*_*i*_ are Fermi velocities given in Supplementary Note 3. For HgTe, we find that the Weyl node located at 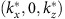 is right-handed, from which the chirality of other Weyl nodes can be derived since all of them are related by symmetry. The locations and chiralities of the eight ideal Weyl nodes are shown in Fig. 2.

One hallmark of Weyl semimetal is the existing of topologically protected surface Fermi arcs. In the ideal Weyl semimetal phase, the electronic states in the *k*_*z*_=0, *k*_*x*_=*k*_*y*_ and *k*_*x*_=−*k*_*y*_ planes are all gapped so that the *Z*_2_ topological invariant in any of these two-dimensional time-reversal-invariant subsystems is well-defined; these *Z*_2_ invariants are all nontrivial because of the band inversion at the Γ point. Consequently, these subsystems carry gapless helical edge modes, which have important implications to possible Fermi arc patterns. Combined with the known chirality of different Weyl nodes, it is expected that the Fermi arcs form a closed circle in (001) surface Brillouin zone (BZ) and that there are open Fermi arcs on the (100) or (010) surface BZ.

### The first-principles calculations

To confirm the results predicted in the effective **k·p** theory above, we carry out first-principles calculations on HgTe. For the compressive in-plane strain with *a*=0.99*a*_0_ and *c*=1.02*a*_0_, while the fourfold degeneracy of the Γ_8_ band at the Γ point is lifted to open a gap, the two intermediate bands touch at eight discrete points: 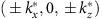 and 
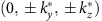
 with 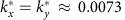
Å^−1^ and 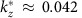
Å^−1^, schematically shown in Fig. 2. These eight gapless points are exactly the Weyl nodes predicted by the effective **k**·**p** model above. Importantly, these Weyl nodes all exactly locate at the Fermi level without coexisting with any trivial bands. It precisely realizes an ideal Weyl semimetal! On the contrary, with the tensile strain with *a*=1.01*a*_0_ and *c*=0.98*a*_0_, a full band gap opens in the entire BZ, which results in a strong topological insulator because of the band inversion between the Γ_6_ and Γ_8_ bands. Qualitative difference between tensile and compressive in-plane strains in HgTe is due to the different response of *p*_*x,y*_ and *p*_*z*_ orbitals to the strain.

**Figure 3:**
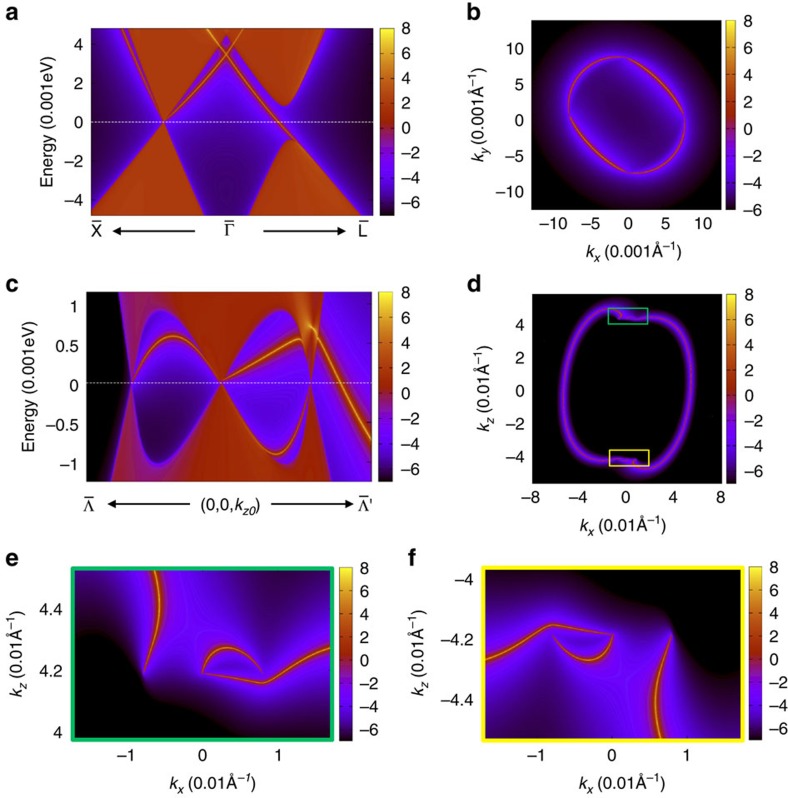
Surface states and Fermi arcs. Electronic structure of HgTe with surfaces in the ideal Weyl semimetal phase. (**a**) Band structure projected onto the (001) surface BZ. The red lines denote the surface states. (**b**) Surface Fermi arcs in the (001) surface BZ form a closed loop, because each gapless point has the monopole charge of +2 or −2. (**c**) Band structure projected onto the (010) surface BZ. (**d**) Surface Fermi arcs in the (010) surface BZ. (**e**,**f**) To see Fermi arcs clearly, the Fermi surfaces around the Weyl nodes are zoomed in.

A key feature of the Weyl semimetal is its unusual surface Fermi arcs, which start from one Weyl node and end at another with opposite chirality^2^. Figure 3a shows the projected bands along 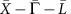 in the (001) surface BZ and surface states can be seen clearly. As the gapless point 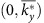 in the 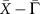 line, projected from the two Weyl nodes 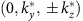, has monopole charge −2, two Fermi arcs have to connect to this point. Similar consideration can be applied to other gapless points 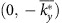 and 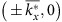 in the (001) surface BZ. The Fermi arcs form a closed loop, as shown in Fig. 3b, which is consistent with the nontrivial *Z*_2_ topological invariant defined in the two-dimensional planes of *k*_*x*_=±*k*_*y*_. Along the bulk-gapped line of 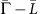, there is a single edge mode across the Fermi level, consistent with the topological properties of Fermi arcs forming a loop on the (001) surface BZ.

The Fermi arcs on the (010) surface is even more interesting. From the projected bands shown in Fig. 3c, we can see that a single chiral edge mode comes out of the gapless points 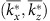 and two chiral edge modes come out of the gapless point 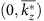, which are consistent with their monopole charges +1 and −2, respectively. The corresponding Fermi surface is shown in Fig. 3d, and the marked regions are zoomed-in in Fig. 3e,f, where discontinuous Fermi arcs are clearly seen. The Fermi arc originating from the point 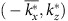 and terminating at the point 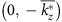 spans across a significant portion of the BZ (about 9% of reciprocal lattice constant), which is of great advantage for experimental detection such as angle resolved photoemission spectroscopy (ARPES). The Fermi arc pattern may change upon varying chemical environment on the surface, but the parity of number of Fermi arcs is topologically stable.

**Figure 4:**
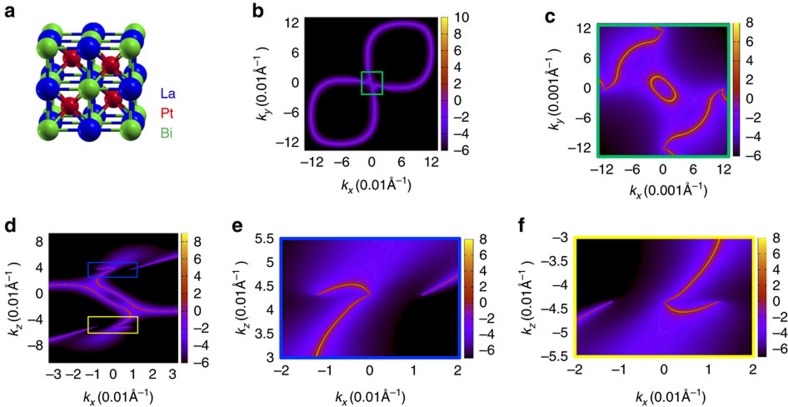
Surface states and Fermi arcs of strained LaPtBi. Electronic structure of LaPtBi with surfaces in the ideal Weyl semimetal phase. (**a**) The stuffed zinc-blende structure of LaPtBi. (**b**) Surface Fermi arcs in the (001) surface BZ also forms a closed loop, though the pattern is different from that of HgTe. (**c**) The surface states around 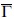 are zoomed in. (**d**,**e**,**f**) Fermi surfaces in the (010) surface BZ exhibit Fermi arcs in **d**, and the marked regions are zoomed-in in **e** and **f**, respectively.

The analysis of realizing ideal Weyl fermions in strained HgTe leads to a general guiding principle to search for ideal Weyl semimetals in noncentrosymmetric materials with nontrivial band inversion. Noncentrosymmetric half-Heusler compounds with band inversion are another family of such materials which could also realize ideal Weyl semimetals under a compressive in-plane strain. For illustration, we perform first-principles calculations on LaPtBi (Fig. 4a) with a compressive in-plane strain of *a*=0.99*a*_0_ and *c*=1.02*a*_0_. Indeed, it also shows eight ideal Weyl nodes at 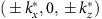 and 
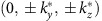
 with 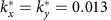
Å^−1^ and 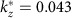
Å^−1^. Similar to HgTe, the surface Fermi arcs also forms a closed loop in the (001) surface BZ, as shown in Fig. 4b,c. The Fermi arcs in the (010) surface are shown in Fig. 4d, whose marked regions are zoomed-in in Fig. 4e,f.

## Discussion

We have shown that applying in-plane strain in HgTe-class materials can give rise to ideal Weyl semimetals with only four pairs of Weyl nodes exactly locating at the Fermi level. Experimentally, such strain in the *xy* plane may be effectively obtained by growing samples on substrates with a smaller lattice constant. For example, materials with zinc-blende structure such as GaSb, InAs, CdSe, ZnTe and HgSe are promising substrates for growing HgTe to achieve the desired in-plane compressive strain. The locations of Weyl nodes for various strength of strains in HgTe and LaPtBi are calculated and summarized in Supplementary Table 1 (Supplementary Note 2). The predicted Weyl nodes and surface Fermi arcs can be directly verified by ARPES measurements. Moreover, transport experiments can be used to detect unusual bulk magneto-transport properties induced by the chiral anomaly of ideal Weyl fermions^27^.

Ideal Weyl semimetals predicted in the strained HgTe-class materials are interesting on their own. It further provides a perfect platform to study the interplay between ideal Weyl fermions and other exotic phenomena, especially in half-Heusler compounds, including superconductivity in LaPtBi (ref. 43), magnetism in GdPtBi, and heavy fermion behaviour in YbPtBi (ref. 44). Interestingly, space-time supersymmetry may also emerge at the superconducting quantum critical point in ideal Weyl fermions^31^. This class of ideal Weyl semimetals thus opens a broad avenue for fundamental research of emergent physics as well as potential applications of low-power quantum devices.

## Methods

### The first-principles calculations

The first-principle calculations are carried out in the framework of the Perdew–Burke–Ernzerhof-type generalized gradient approximation of the density functional theory through employing the BSTATE package^45^ with the plane-wave pseudo-potential method. The kinetic energy cutoff is fixed to 340 eV, and the **k**-point mesh is taken as 16 × 16 × 16 for the bulk calculations. The spin-orbit coupling is self-consistently included. The experimental lattice constants are used with *a*_0_=6.46 Å for HgTe and 6.829 Å for LaPtBi. In order to simulate the uniaxial strain along the [001] direction, we fix the experimental volume but change the ration *a*/*c*, where *a* is the lattice constant in the *x*–*y* plane and *c* is the lattice constant along *z* axis (the [001] direction). Without loss of generality, we take the parameters *a*=0.99*a*_0_ and *c*=1.02*a*_0_ for the tensile strain and *a*=1.01*a*_0_ and *c*=0.98*a*_0_ for the compressive strain. To exhibit unique surface states and Fermi arcs on the surface, we employ maximally localized Wannier functions^46^,^47^ to first obtain the *ab initio* tight-binding model of the bulk HgTe and LaPtBi and then pick these bulk hopping parameters to construct the tight-binding model of the semi-infinite system with the (001) or (010) surface as the boundary. The surface Green's function of the semi-infinite system, whose imaginary part is the local density of states to obtain the dispersion of the surface states, can be calculated through an iterative method^48^.

## Additional information

**How to cite this article:** Ruan, J. *et al*. Symmetry-protected ideal Weyl semimetal in HgTe-class materials. *Nat. Commun.* 7:11136 doi: 10.1038/ncomms11136 (2016).

## Supplementary Material

Supplementary Information Supplementary Figures 1-2, Supplementary Tables 1, Supplementary Notes 1-3 and Supplementary References
