# Supplementary material for: Symmetry-protected ideal Weyl semimetal in HgTe-class materials
Source: Nat Commun. 2016 Apr 1;7:11136. doi: 10.1038/ncomms11136 (PMC4822222; doi:10.1038/ncomms11136)
Supplement: Supplementary Information — Supplementary Figures 1-2, Supplementary Tables 1, Supplementary Notes 1-3 and Supplementary References [file ncomms11136-s1.pdf]

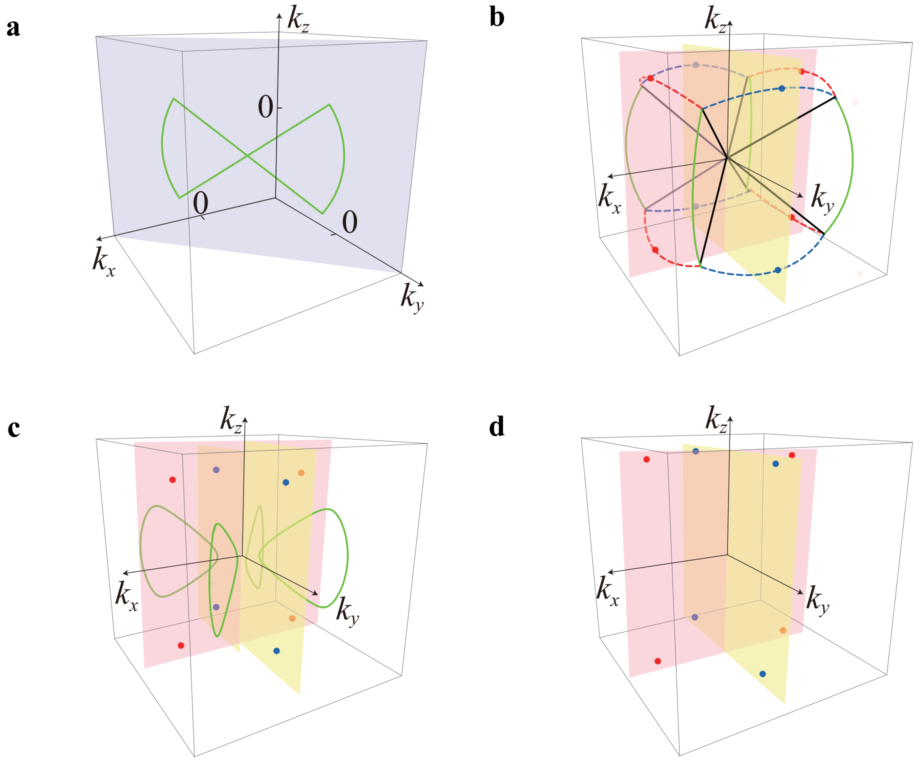

Supplementary Figure 1: | **The characterization of band crossings in HgTe with and without applied strain.** (**a**) The green line represents the line nodes in the $k_x = -k_y$ mirror plane of HgTe without strain. (**b**) The line node structure without strain in the full bulk BZ. The blue, red, and green lines represent line nodes lying in the mirror plane but not along any diagonal direction. The black solid lines label line nodes in diagonal directions. The red (blue) dashed lines cross the $k_y = 0$ ($k_x = 0$) plane where the crossing points are indicated by red (blue) points. These dashed line nodes, except those crossing points, split once strain is applied due to the breaking of their mirror symmetries. (**c**) After applying a small strain, the line nodes in the $k_x = \pm k_y$ mirror planes gradually shrink, as shown by the green lines. The red and blue points represent the Weyl nodes in the $k_x = 0$ and $k_y = 0$ planes, protected by C_{2T} symmetry. (**d**) When the compressive strain exceeds a critical value, i.e. $\delta < \delta_c^{\mathrm{Weyl}}$, there are no line nodes any more and the Weyl nodes, indicated by the red and blue points, are type-I such that they are all located exactly at the Fermi level and the system is in the ideal Weyl semimetal phase.

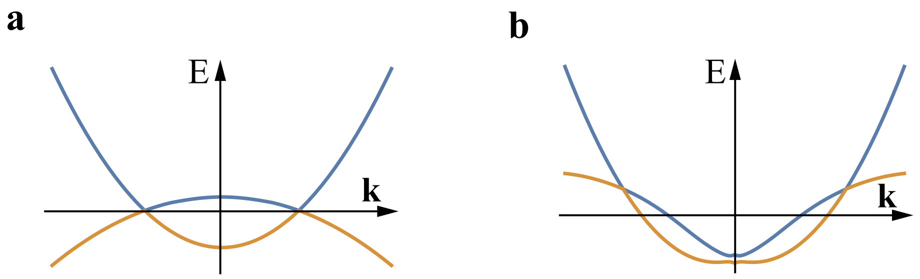

Supplementary Figure 2: | **Schematic dispersions of the two types of Weyl nodes.** (**a**) ideal Weyl semimetals and (**b**) type-II Weyl semimetals.

Supplementary Table 1: The location of Weyl nodes $(k_x^*, 0, k_z^*)$ under different in-plane strains for (a) HgTe and (b) LaPtBi. Here a and c denote the lattice constants after applying the in-plane strain, and a_0 denotes the experimental lattice constant of HgTe or LaPtBi.

(a) HgTe

a/a_0	c/a_0	$k_x^*(\text{Å}^{-1})$	$k_z^*(\text{Å}^{-1})$
0.995	1.01	.0069	.0294
0.990	1.02	.0073	.0417
0.985	1.03	.0077	.0513
0.980	1.04	.0080	.0593
0.976	1.05	.0085	.0671
0.971	1.06	.0086	.0737

(b) LaPtBi

a/a_0	c/a_0	$k_x^*(\text{Å}^{-1})$	$k_z^*(\text{Å}^{-1})$
0.995	1.01	.0098	.0306
0.990	1.02	.0133	.0434
0.985	1.03	.0166	.0541
0.980	1.04	.0198	.0639
0.976	1.05	.0231	.0729
0.971	1.06	.0267	.0817

Supplementary Note 1: Systematic evolution of the band structure under strain
As mentioned in the main text, the HgTe-class materials which carry T_d point group symmetry, do not respect inversion symmetry. The inversion asymmetry in these systems results in qualitatively different band structures compared to those with inversion symmetry, e.g., α-Sn. Instead of a simple quadratic band touching point (at Γ) exactly at Fermi level, two intermediate bands of the four Γ_8 bands in HgTe-class materials touch along a line node in the six mirror planes. These line nodes are protected by mirror symmetry even though they are generically away from the Fermi level. As a consequence, the HgTe-class materials have small electron and hole pockets, even at stoichiometry.

To illustrate these line nodes, we diagonalize Hamiltonian in the mirror plane, e.g., the $k_x = -k_y$ plane as shown by the shaded plane in Supplementary Figure 1**a**. The unstrained Hamiltonian is given in the main text, i.e., $\mathcal{H}_{\text{unstrained}} = \mathcal{H}_{\text{Luttinger}} + \mathcal{H}_{\text{BIA}}$. For simplicity, only the linear term in $\mathcal{H}_{\text{BIA}}$ is taken into consideration and higher-order terms in $\mathcal{H}_{\text{BIA}}$ won't affect the results we obtain in this section qualitatively. The line nodes lying in the $k_x = -k_y$ mirror plane is given by the following equation:

$$2(c_1^2 - c_2^2)k_z^2 - 3|\alpha^2 - 2c_1^2k_z^2| + (2c_2^2 - 8c_1^2)k_y^2 + 3\alpha^2 = 0. \quad (1)$$

where c_i is defined in Supplementary Note 3. This bow-shape line node is shown in Supplementary Figure 1**a** by a green line. There are six symmetry-related mirror planes and each of them contains such a bow-shape line node. Two line nodes overlap each other along the diagonal direction where their mirror planes intersect, as indicated by the black lines in Supplementary Figure 1**b**. Since the system has a C_3 symmetry along the diagonal direction, the two intermediate bands form a two-dimensional irreducible representation that explains the overlaps of two line nodes along this direction. The red , blue or green lines in Supplementary Figure 1**b** indicate the parts of the line nodes not along the diagonal directions.

Upon applying strain in the xy plane, the crystalline symmetry of the material is lowered from T_d to D_{2d}. The mirror symmetries in the $k_x = \pm k_z$ and $k_y = \pm k_z$ planes are broken, while those in $k_x = \pm k_y$ planes survive. As a result, for small strain the line nodes located at $k_x = \pm k_y$ plane survive as they are protected by the unbroken mirror symmetry, even though they contract as the applied strain increases. The evolution of the line nodes is shown in Supplementary Figure 1**c** where the closed green lines indicate the shrinking line nodes under a small strain. When the applied strain exceeds critical value, these line nodes eventually disappear.

On the other hand, the line nodes in $k_x = \pm k_z$ and $k_y = \pm k_z$ planes are split immediately , upon applying strain except the eight discrete points. These eight discrete points are the intersecting points between the $k_y = 0$ ($k_x = 0$) plane and the line nodes in the $k_x = \pm k_z$ ($k_y = \pm k_z$) planes, as shown by red or blue color points in Supplementary Figure 1**b**. As mentioned in the main text, these eight discrete points are protected from gapping out by $C_{2T} \equiv C_2 \cdot T$ symmetries. Applying strain only gradually shift these points in the $k_y = 0$ or $k_x = 0$ planes. Supplementary Figure 1(c) shows these discrete points upon applying a small strain. These eight discrete points are actually Weyl points, which reside in the $k_x = 0$ or $k_y = 0$ planes protected by the C_{2T} symmetry (see Supplementary Note 3 for details).

When the strain is sufficiently small but finite, these points are type-II Weyl points. These type-II Weyl points in the $k_y = 0$ plane shift to $|k_z| > |k_x|$ region if compressive strain is applied while they shift to $|k_z| < |k_x|$ region if tensile strain is applied, and Weyl points in the $k_x = 0$ have the similar behavior related by symmetry. Thanks to this qualitatively different response between tensile and compressive strain, the system evolve into different phases at sufficiently large strain. When the tensile strain increases, these type-II Weyl points first move to the k_x or k_y axis in the $k_z = 0$ plane, then move within the $k_z = 0$ plane (since there is C_{2T} symmetry in the $k_z = 0$ plane), and finally they annihilate one another with opposite chiralities in $k_z = 0, k_x = \pm k_y$ lines when the tensile strain reaches a critical value. When the strain exceeds the critical value, the system enters into a strong topological insulator phase with nontrivial Z_2 topological-invariant.

On the other hands, when the compressive strain increases from zero, the eight type-II Weyl nodes shift towards a larger $|k_z|$ direction. When the compressive strain exceeds a critical value, all trivial Fermi surface vanishes and the eight type-II Weyl nodes evolve to type-I ideal Weyl nodes located exactly at the Fermi level, as shown in Supplementary Figure 1**d**.

Supplementary Note 2: The Weyl nodes under different strains
In this section, we calculate locations of ideal Weyl nodes for different in-plane compressive strains for both HgTe and the half-Heusler compound LaPtBi, as shown in Supplementary Table 1. The Weyl nodes move slowly towards larger momentum points in both k_x and k_z directions for increasing strain. As the Weyl nodes can only be pair-annihilated in the $k_z = \pi$ plane, the slow motion of Weyl nodes with increasing strain indicates that the ideal Weyl semimetal phase is stable under a broad range of strain. Indeed, for the large strain of $a/a_0 = 0.971$ and $c/a_0 = 1.06$, the Weyl nodes are still far away from the $k_z = \pi$ plane. Moreover, the larger separation between Weyl nodes in momentum

space under increasing in-plane compressive strain can make the observation of them by Angle resolved photoemission spectroscopy (ARPES) experiments easier.

Supplementary Note 3: The effective k·p theory at finite strain
In this section, we use following Gamma matrices [1]:

$$\Gamma^1 = \frac{1}{\sqrt{3}}\{J_y, J_z\},\ \Gamma^2 = \frac{1}{\sqrt{3}}\{J_z, J_x\},\ \Gamma^3 = \frac{1}{\sqrt{3}}\{J_x, J_y\}, \Gamma^4 = \frac{1}{\sqrt{3}}(J_x^2 - J_y^2),\ \Gamma^5 = J_z^2 - \frac{5}{4} \tag{2}$$

to write the Luttinger Hamiltonian:

$$\mathcal{H}_{\text{Luttinger}}(\mathbf{k}) = c_0\mathbf{k}^2 + c_1\sum_{i=1}^{3} d_i\Gamma^i + c_2\sum_{i=4}^{5} d_i\Gamma^i, \tag{3}$$

where $d_1(\mathbf{k}) = \sqrt{3}k_yk_z,\ d_2(\mathbf{k}) = \sqrt{3}k_xk_z,\ d_3(\mathbf{k}) = \sqrt{3}k_xk_y,\ d_4(\mathbf{k}) = \frac{\sqrt{3}}{2}(k_x^2 - k_y^2),\ d_5(\mathbf{k}) = \frac{1}{2}(2k_z^2 - k_x^2 - k_y^2)$ and $c_0 = \alpha_0 + \frac{5}{4}(\alpha_1 + \alpha_2),\ c_1 = \alpha_1,\ c_2 = (\alpha_1 + \alpha_2)$.

Now, we explore the behavior of HgTe under sufficiently large strain (exceeds the critical value g_c^{Weyl}) using the effective **k·p** theory. From Clebsch-Gordan coefficients, we know

$$|\frac{3}{2}, \frac{3}{2}\rangle = |p_x + ip_y, \uparrow\rangle,\ |\frac{3}{2}, -\frac{3}{2}\rangle = |p_x - ip_y, \downarrow\rangle, \tag{4}$$

$$|\frac{3}{2}, \frac{1}{2}\rangle = \sqrt{\frac{1}{3}}|p_x + ip_y, \downarrow\rangle + \sqrt{\frac{2}{3}}|p_z, \uparrow\rangle,\ |\frac{3}{2}, -\frac{1}{2}\rangle = \sqrt{\frac{1}{3}}|p_x - ip_y, \uparrow\rangle + \sqrt{\frac{2}{3}}|p_z, \downarrow\rangle, \tag{5}$$

where $|\frac{3}{2}, J_z\rangle$ labels the wave function of a single electron with $J_z = \pm\frac{3}{2}, \pm\frac{1}{2}$. Electrons with $J_z = \pm 3/2$ have definite orbital angular momentum $l_z = \pm 1$ while electrons with $J_z = \pm 1/2$ are superpositions of the wave functions with $l_z = \pm 1$ and $l_z = 0$. Since compressive strain in the xy plane shortens the lattice distance along the x and y directions, the wave functions with orbital angular momentum $l_z = \pm 1$ get more overlapped than that of $l_z = 0$, and their energy shift is larger than that of $l_z = 0$. This indicates that the main effect induced by the strain in the xy plane is given by the following perturbation:

$$\mathcal{H}_{\text{strain}} = -g(J_z^2 - \frac{5}{4}) = -g\Gamma^5, \tag{6}$$

where g is related to the strength of applied strain δ. Moreover, we obtain $g < 0$ for compressive strain ($\delta < 0$) and $g > 0$ for tensile strain ($\delta > 0$).

We shall focus on the case that $g < g_c^{\text{Weyl}}$ here. For this case, as explained in the main text, treating $\mathcal{H}_0 \equiv \mathcal{H}_{\text{Luttinger}} + \mathcal{H}_{\text{strain}}$ as unperturbed Hamiltonian and the BIA part $\mathcal{H}_{\text{BIA}}$ as perturbation is a better way to characterize the band features around Weyl nodes. The quadratic touching point in Luttinger Hamiltonian $\mathcal{H}_{\text{Luttinger}}$ is split into two Dirac points locating at k_z axis for compressive strain because $\mathcal{H}_{\text{strain}}$ has only D_{4h} symmetry which is lower than the O_h symmetry of the Luttinger Hamiltonian. Specifically, the dispersion is given by $E = c_0k^2 \pm \sqrt{c_1^2(d_1^2 + d_2^2 + d_3^2) + c_2^2d_4^2 + (c_2d_5 + g)^2)}$, leading to two Dirac points at $(0, 0, \pm\sqrt{g/c_2})$. Expanding the Hamiltonian around the touching point $(0, 0, \sqrt{g/c_2})$, we obtain the Dirac Hamiltonian:

$$\mathcal{H}_{\text{Dirac}}(\mathbf{k}) \equiv v_z'k_z + v_\perp(k_y\Gamma^1 + k_x\Gamma^2) + v_zk_z\Gamma^5, \tag{7}$$

where $v_z' = 2c_0\sqrt{\frac{g}{c_2}}, v_\perp = -\sqrt{\frac{3gc_1^2}{c_2}}, v_z = -2\sqrt{gc_2}$. These Dirac points are protected by inversion symmetry, time reversal symmetry, as well as the $S_4(z)$ symmetry of $\mathcal{H}_0$. In HgTe, inversion symmetry is actually broken which has important consequences even though the breaking is weak. As a result of BIA, linear, cubic, as well as higher-order terms compatible with the T_d symmetry are allowed in the Hamiltonian and we treat them as perturbations.

We first consider the effect of the linear term in $\mathcal{H}_{\text{BIA}}$[2]:

$$\mathcal{H}_{\text{linear}} = \alpha\Big[(\frac{-\sqrt{3}}{2}\Gamma^{15} + \frac{3}{2}\Gamma^{14})k_x + (\frac{-\sqrt{3}}{2}\Gamma^{25} - \frac{3}{2}\Gamma^{24})k_y + \sqrt{3}\Gamma^{35}k_z\Big]. \tag{8}$$

For simplicity, we approximate $\mathcal{H}_{\text{linear}}$ by its form at the Dirac point $(0, 0, \sqrt{g/c_2})$ and denote it as $\mathcal{H}_{\text{m}} = m\Gamma^{35}$, where $m = \alpha\sqrt{3g/c_2}$. In the presence of $\mathcal{H}_{\text{m}}$, the low-energy dispersion is given by $E_k = v_z'k_z \pm \sqrt{(v_\perp\sqrt{k_x^2 + k_y^2} \pm m)^2 + v_z^2k_z^2}$.

It is easy to see that the Dirac points are split and lead to two doubly degenerate line nodes satisfying the two equations: $k_x^2 + k_y^2 = (m/v_\perp)^2$ and $k_z = \pm\sqrt{g/c_2}$. In general, line nodes in 3D momentum space are not stable against further generic perturbations. For instance, the cubic term in $\mathcal{H}_{\rm BIA}$ given by $\mathcal{H}_{\rm cubic} = \beta(\{k_x, k_y^2 - k_z^2\}J_x + {\rm cp})$, where β is a constant that describes the strength of cubic term, can split the line nodes. Interestingly, eight discrete gapless points, which are the crossing points of original line nodes with the $k_x = 0$ or $k_y = 0$ plane, survive from generic perturbations in $\mathcal{H}_{\rm BIA}$. These stable gapless discrete points in the $k_x = 0$ or $k_y = 0$ plane are protected by a special symmetry $\mathrm{C}_{2T} = \mathrm{C}_2 \cdot T$ that forms little group of these planes which we shall explain below.

We now consider the $k_y = 0$ plane to explain how its C_{2T} symmetry can protect a gapless Weyl point in this plane. The $k_y = 0$ plane respects the C_{2T} symmetry which is given by

$$\mathrm{C}_{2T} \propto e^{-i\pi J_y} \cdot e^{i\pi J_y} K = K, \tag{9}$$

where K is the complex conjugation operator, an anti-unitary transformation. After expanding the Hamiltonian, i.e., $\mathcal{H}_{\rm Dirac} + \mathcal{H}_m$, at one of the four crossing points $(\frac{m}{v_\perp}, 0, \sqrt{\frac{g}{c_2}})$ in the $k_y = 0$ plane, we obtain an effective Hamiltonian:

$$\mathcal{H}(\mathbf{k}) = v_z' k_z + v_\perp(k_x\Gamma^2 + k_y\Gamma^1) + v_z k_z \Gamma^5 + m(\Gamma^2 + \Gamma^{35}). \tag{10}$$

Since two intermediate bands of four bands touches, we implement a unitary transformation U that diagonalizes Γ^2 and Γ^{35} simultaneously to project the Hamiltonian to those bands. The resultant Hamiltonian in the $k_y = 0$ plane reads ($\mathcal{H}_U = U\mathcal{H}U^\dagger$):

$$\mathcal{H}_U(k_y = 0) = \begin{pmatrix} -v_\perp k_x - 2m & v_z k_z & 0 & 0 \\ v_z k_z & v_\perp k_x + 2m & 0 & 0 \\ 0 & 0 & v_z k_z & -v_\perp k_x \\ 0 & 0 & -v_\perp k_x & -v_z k_z \end{pmatrix}, \tag{11}$$

which has a simple block-diagonal form. It is easy to see that the low-energy sector is given by second block and it can be written compactly as:

$$\mathcal{H}_U'(k_y = 0) = v_z k_z \sigma^z - v_\perp k_x \sigma^x. \tag{12}$$

Moreover, the C_{2T} operator does not change after this unitary transformation, i.e. $U\mathrm{C}_{2T}U^\dagger = \mathrm{C}_{2T} \propto K$. An immediate consequence is that the gapless point locating at $k_y = 0$ plane is protected: any gap-opening term proportional to σ^y is not allowed because it breaks the C_{2T} symmetry. Namely, because the Hamiltonian respects this special C_{2T} symmetry, the eight Weyl points in the $k_x = 0$ and $k_y = 0$ planes are stable against all possible BIA and strain perturbations if they are not too strong. These perturbations can only shift these Weyl points in the $k_x = 0$ and $k_y = 0$ plane.

We now include the cubic term $\mathcal{H}_{\rm cubic} \equiv \beta[k_x(k_y^2 - k_z^2)J_x + \text{c.p.}]$ in $\mathcal{H}_{\rm BIA}$ to obtain the effective Hamiltonian of the Weyl points with dispersions away from the C_{2T}-planes. Expanding it at the gapless point $(\frac{m}{v_\perp}, 0, \sqrt{\frac{g}{c_2}})$, we obtain a linear term in k_y: $\frac{2}{\sqrt{3}} v_y J_y k_y$, where J_i is the angular momentum operator, and $v_y = \sqrt{3}\beta\left(\frac{g}{c_2} - (\frac{m}{v_\perp})^2\right) = \sqrt{3}\beta(\frac{g}{c_2} - \frac{\alpha_2^2}{c_1^2})$. Projecting this linear terms into the low-energy subspace of two intermediate bands around the gapless Weyl point, we obtain the Weyl Hamiltonian:

$$\mathcal{H}_{\rm Weyl} = \sum_i v_i k_i \sigma^i. \tag{13}$$

where $v_x = \sqrt{\frac{3g\alpha_1^2}{\alpha_1+\alpha_2}}$, $v_y = -\sqrt{3}\beta(\frac{g}{\alpha_1+\alpha_2} - \frac{\alpha_2^2}{\alpha_1^2})$, and $v_z = -2\sqrt{g(\alpha_1+\alpha_2)}$ and it is exactly the Weyl Hamiltonian appeared in the main text. For HgTe, we find that the velocity v_y is negative because $\beta > 0$. So this Weyl node is right-handed as mentioned in the main text.

Supplementary References

[1] Murakami, S., Nagosa, N. & Zhang, S.-C. SU(2) non-abelian holonomy and dissipationless spin current in semiconductors. *Phys. Rev. B* **69**, 235206 (2004).

[2] Winkler, R. Spin-orbit coupling effects in two-dimensional electron and hole systems. *Springer Tracts in Modern Physiscs: Springer, Berlin, Heidelberg*, p. 191 (2003).
